# Gene expression in meningeal lymphatic endothelial cells following traumatic brain injury in mice

**DOI:** 10.1371/journal.pone.0273892

**Published:** 2022-09-06

**Authors:** Ryo Shimada, Yuki Tatara, Kazuhiko Kibayashi

**Affiliations:** Department of Forensic Medicine, School of Medicine, Tokyo Women’s Medical University, Tokyo, Japan; Istituto per la Ricerca e la Cura del Cancro di Candiolo, ITALY

## Abstract

Meningeal lymphatic vessels transport both the cerebrospinal fluid and interstitial fluid to the deep cervical lymph nodes. Traumatic brain injury (TBI) is accompanied by meningeal injury. We hypothesized that the TBI-induced meningeal injury would damage lymphatic vessels and affect brain function. We observed altered gene expression in meningeal lymphatic endothelial cells (LECs) in a mouse model of TBI. Through flow cytometry–based cell sorting, meningeal LECs were obtained from a mouse model of controlled cortical impact 3 days after TBI. Microarray analysis, real-time polymerase chain reaction assays, and enzyme-linked immunosorbent assays were performed to determine mRNA and protein expression levels in meningeal LECs. The number of meningeal LECs was significantly lower in the injury group than in the sham group 3 days after TBI. Additionally, the mRNA expression of lymphatic vessel endothelial hyaluronan receptor 1 (a specific marker of lymphatic vessels) in meningeal LECs was significantly lower in the injury group than in the sham group. The mRNA and protein expression of FMS-like tyrosine kinase 4 and neuropilin 2 (markers of lymphangiogenesis) in meningeal LECs was significantly higher in the injury group than in the sham group. Our findings indicate that TBI is associated with the impairment of meningeal LECs and meningeal lymphangiogenesis, which implicates lymphatic vessel injury in the pathogenesis of this condition.

## Introduction

Traumatic brain injury (TBI) is accompanied by meningeal injury. Moreover, using post-contrast fluid-attenuated inversion recovery magnetic resonance imaging, meningeal damage has been observed without brain injury in patients with minor TBI [1], which indicates that meningeal lymphatic vessels may also be damaged.

Meningeal lymphatic vessels line the dural venous sinus [2]. This structure is connected to the deep cervical lymph nodes, expresses all markers of lymphatic endothelial cells (LECs), and transports both the cerebrospinal fluid and interstitial fluid [3, 4]. The functions of the lymphatic system include removal of excess fluid from tissues and production of immunocompetent cells (lymphocytes, monocytes, and plasma cells that produce antibodies). The glial lymphatic system in the brain removes waste products accumulated in the brain and has been reported to be activated during sleep [5]. However, it is thought that damage to the meningeal lymphatic vessels may prevent lymphatic fluid from being transported, causing waste products to accumulate in the brain. In a meningeal lymphatic drainage study, the lymphatic dysfunction that was present prior to TBI led to increased neuroinflammation and reduced cognitive function [6]. By analyzing the gene expression in meningeal LECs produced in response to meningeal lymphatic vessel injury, we can identify the genes involved in meningeal lymphatic vessel dysfunction and determine the effects of lymphatic vessel injury on the brain, which may be useful in the treatment of TBI.

Da Mesquita et al. isolated LECs from the meninges using antibodies against CD45-CD31+podoplanin (PDPN)+ cells via flow cytometry [7]. CD45 (protein tyrosine phosphatase receptor type C, PTPRC) is a member of the protein tyrosine phosphatase (PTP) family, which comprises signaling molecules that control various cellular processes such as mitosis, oncogenic transformation, cell growth, and differentiation. CD45 is often used as a leukocyte marker [8]. CD31 (platelet and endothelial cell adhesion molecule 1, PECAM1) is a member of the immunoglobulin superfamily and constitutes most intercellular junctions of endothelial cells [9]. PDPN is a type I endogenous membrane glycoprotein that is expressed in various tissues, such as the choroid plexus and mesothelium [10]. Its functions include regulating cell motility and adhesion [11] and correcting development of the lymphatic vasculature [12].

Lymphatic vessel endothelial hyaluronan receptor 1 (LYVE1) is a marker of LECs and is specifically expressed in meningeal lymphatic vessels [13]. LYVE1, a type I membrane endogenous glycoprotein, binds both soluble and immobilized hyaluronan [14]. LYVE1 can also be expressed by activated myeloid cells that produce functional lymphatic progenitors in the meninges [15]. It is involved in lymphangiogenesis, along with FMS-like tyrosine kinase 4 (FLT4 or VEGFR3), vascular endothelial growth factor C (VEGFC), and neuropilin 2 (NRP2) [16]. VEGFC is a member of the vascular endothelial growth factor/platelet-derived growth factor (VEGF/PDGF) family; it promotes lymphangiogenesis, endothelial cell growth, and angiogenesis. The pro-protein of VEGFC is cleaved into its fully active form, resulting in the binding and activation of VEGFR2 (kinase insert domain receptor, KDR) and the FLT4 receptor (VEGFR3) [17]. *FLT4* encodes a tyrosine kinase receptor for VEGFC and VEGFD, which is associated with the maintenance of the lymphatic endothelium. NRP2, a member of the neuropilin family of receptors, interacts with VEGF [13].

To the best of our knowledge, transcription profiling has not yet been performed on meningeal LECs in a mouse model of TBI. It was hypothesized that the expression of genes specifically expressed in meningeal LECs changes following TBI; accordingly, we determined the expression of these genes in meningeal LECs. Furthermore, because meningeal lymphatic vessels are thought to be repaired after damage, we investigated whether lymphangiogenesis is involved in the repair of meningeal lymphatic vessels.

## Materials and methods

### Laboratory animals and surgical procedures

**Fig 1:**
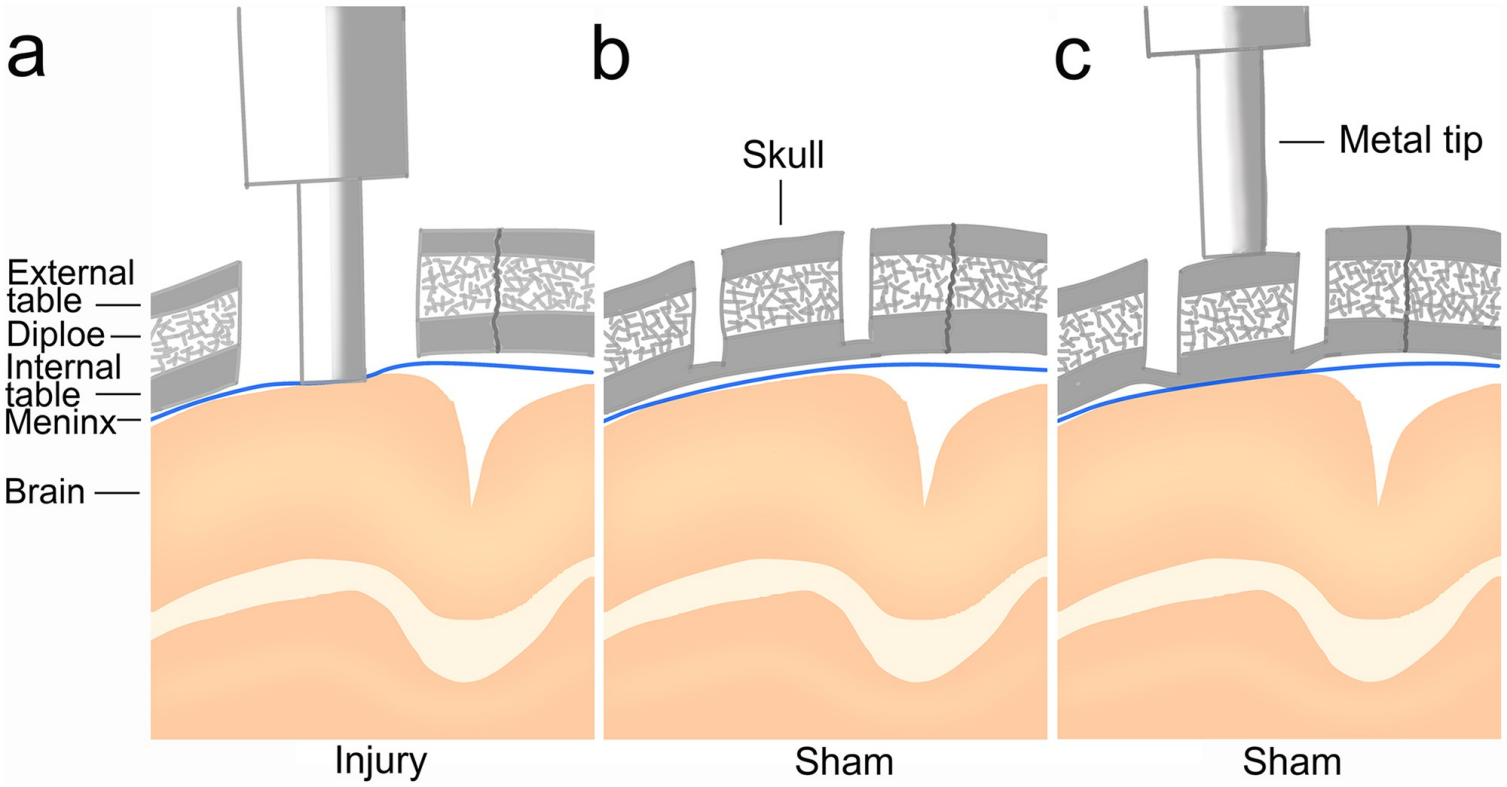
Surgery for the CCI mouse model. (a) In the injury group, the skull was removed, and a rounded metal tip was used to directly impact the brain. (b) For the sham group, resection of the external table and upper part of the internal table of the skull was performed using a drill. (c) The rounded metal tip was pushed into the bone flaps manually to transmit the pressure to the brain via the bone flaps in the sham group.

The procedures for the animal experiments were approved by the Ethical Review Committee of Animal Experiments at Tokyo Women’s Medical University. All mice were housed in a specific pathogen free (SPF) environment with periodic microbial monitoring tests, were kept in a controlled environment with a temperature of 23±1°C and humidity of 50±10%, on a 12-hour light/dark cycle (lights on at 8:00), and had free access to normal rodent feed and sterile tap water. The injury and sham groups were randomly allocated from a total of 120 male C57BL/6J mice weighing 22–25 g (approximately 10 weeks old). An intraperitoneal (i.p.) injection of three types of mixed agents—medetomidine hydrochloride (Domitor; 0.3 mg/kg, ZENOAQ, Nippon Zenyaku Kogyo Co. Ltd., Fukushima, Japan), midazolam (4 mg/kg, Fuji Parma Co. Ltd., Toyama, Japan), and butorphanol (5 mg/kg, Meiji Seika Pharma Co. Ltd., Tokyo, Japan)—was used for mice anesthetization [18]. Following anesthetization, 4% isoflurane (Wako Pure Chemical Industries, Co. Ltd., Osaka, Japan) was delivered using an inhalation anesthesia apparatus and an enclosed chamber (Narcobit-E; Natsume Seisakusho, Tokyo, Japan) [19]. While rectal temperature was maintained at 37°C using a warming pad with a feedback probe that was connected to a small animal warmer and thermometer (Bio Research Center Co., Ltd., Aichi, Japan), the mice were placed in a stereotaxic frame, and their scalps were shaved and cut open to expose the skull. To open the bony window of the skull for controlled cortical impact (CCI) treatment, a craniotomy 5 mm in diameter over the left parietal cortex was performed using a dental trephine drill, which was located 6 mm posterior to the coronal suture and 6 mm lateral to the sagittal suture. A CCI device (Amscien Instruments LLC, Richmond, VA, USA) was used to induce TBI in the mice of the injury group according to the settings of the CCI device in our previous study (Fig 1a) [20]. A pneumatic piston with a 3 mm-diameter rounded metal tip was aligned vertically at 15° over the center of the craniotomy so that the tip was perpendicular to the dural surface at impact and a deformation depth of 2 mm below the dura. To seal the craniotomy, the bone flap adhering to a plastic plate (7 mm diameter, 0.2 mm thickness) was restored, and then, the scalp was sutured closed immediately after CCI treatment. Following i.p. injection of Antisedan (0.3 mg/kg, ZENOAQ) to antagonize Domitor [21], the mice were placed in a heat-controlled cage for maintenance of their body temperature until they recovered from the anesthesia to alleviate suffering.

Although various surgical methods have been used for sham groups [22, 23], in this study, to prevent brain and meninx injury and bleeding caused by the device due to craniotomy, at the same site of the skull drilled as in the injury group, we resected the external table, diploe, and upper parts of the internal table of the bone by drilling and left the bone flap on the surface of the cerebrum without removal [22]. This means that the bone flap is not cut from the skull and is connected to the entire skull by the lower part of the internal table; however, it can be moved sufficiently by pushing. Next, the rounded metal tip was pushed into the bone flaps manually to transmit the pressure to the brain via bone flaps; however, the brain and meninx did not receive CCI treatment (Fig 1b and 1c).

### Sample preparation

Under deep anesthesia using 3% isoflurane (Wako Pure Chemical Industries), which was delivered using the facemask of an inhalation anesthesia apparatus (Narcobit-E) and 150 mg/kg pentobarbital (i.p.), the mice were released to bleed for sacrifice and transcardially perfused with phosphate-buffered saline (PBS, pH 7.4) 3 days after surgery. Since the meninge is small, the meninges from six mice were pooled and used as one sample (*n* = 1) for analysis. For example, *n* = 5 × 6 mice per group, totaling 60 samples for mRNA analysis of the injury and sham groups, and another n = 5 × 6 mice per group, totaling 60 samples for protein analysis of the injury and sham groups; hence, 120 mice were used in this experiment.

For meningeal collection, the muscle was stripped from the skull after scalp incision and the skullcap was removed using surgical scissors and stored in PBS at 4°C until the next step. Subsequently, the meninges (arachnoid mater and dura mater) were carefully exfoliated from the skullcaps using forceps [7].

### Sorting of meningeal LECs using flow cytometry and total RNA and protein extraction

**Fig 2:**
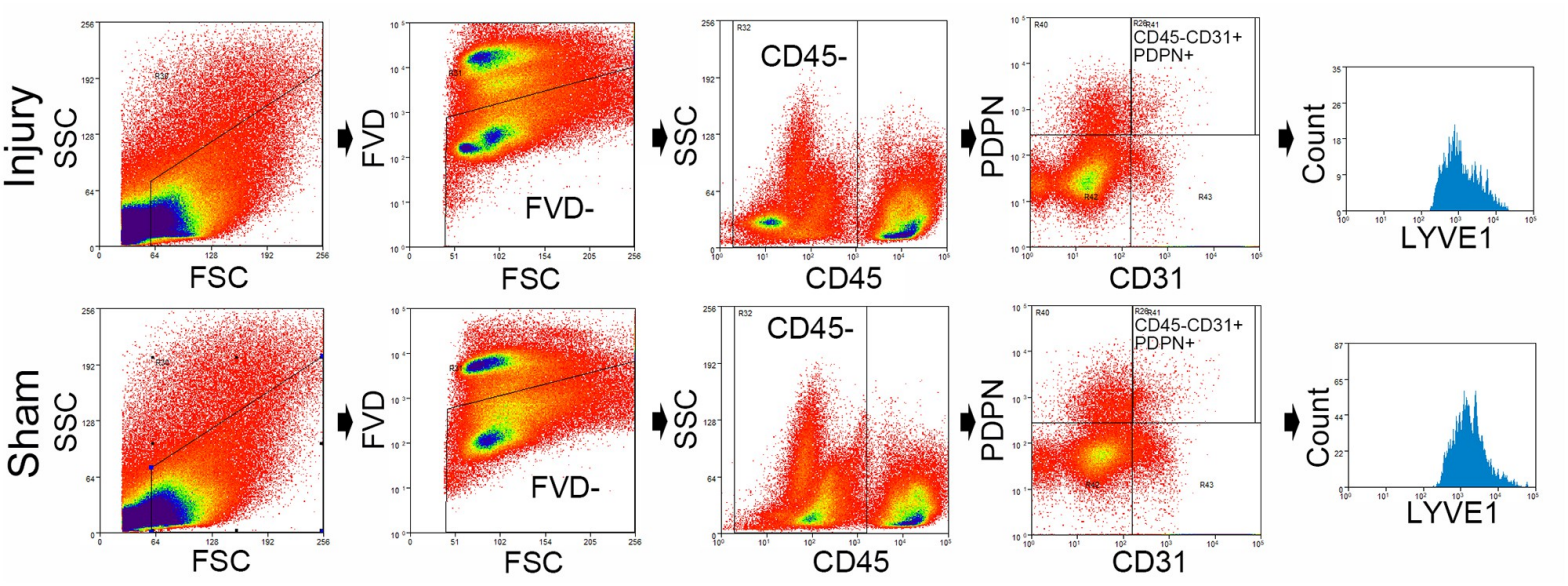
Isolation of meningeal lymphatic endothelial cells (LECs) via flow cytometry. Representative dot and contour plots showing the gating strategy used to isolate LECs via flow cytometry from the meninges of mice with traumatic brain injury and the sham mice. The cell count graph shows the number of cells, which were gated by CD45−CD31+PDPN+, labeled with the anti-LYVE1 antibody.

Individual samples, consisting of six pooled meninges, were digested into cell suspensions for 20 min at 37°C with 1.4 U/mL collagenase VIII from *Clostridium histolyticum* (Sigma-Aldrich, Tokyo, Japan) and 35 U/mL DNase I (Qiagen, Tokyo, Japan) in complete media consisting of Dulbecco’s modified Eagle’s medium/F-12, 4-(2-hydroxyethyl)-1-piperazineethanesulfonic acid (Thermo Fisher Scientific Inc., Tokyo, Japan) supplemented with 2% fetal bovine serum (ScienCell Research Laboratories Inc., Carlsbad, USA) and 1% penicillin-streptomycin, liquid (Thermo Fisher Scientific Inc). After filtration through a 70 μm nylon mesh cell strainer (Falcon, Corning, NY, USA), the cell pellets were washed and resuspended in PBS containing 1 mM ethylenediaminetetraacetic acid (EDTA) and 0.5% bovine serum albumin (BSA). The washed cell pellets were resuspended in PBS containing 1 mM EDTA, 0.5% BSA, eBioscience Fixable Viability Dye eFluor 450 (FVD450, 1:500, Thermo Fisher Scientific Inc.), anti-CD45-BB515 (1:100, clone 30-F11, BD Biosciences, Tokyo, Japan), anti-CD31-Alexa Fluor 647 (1:100, clone 390, BD Biosciences), and anti-PDPN-PE (1:100, clone 8.1.1, eBioscience, Thermo Fisher Scientific Inc.). Furthermore, anti-LYVE1-PE-Cyanine7 (1:100, ALY7, eBioscience, Thermo Fisher Scientific Inc.) was added to label the cells and confirm the purity of meningeal LECs that were collected by the cell sorter. The cells were incubated for 30 min at 4°C and subsequently washed and resuspended in PBS containing 1 mM EDTA and 0.5% BSA. The representative flow plots were employed for meningeal LEC sorting experiments. Using a flow cytometry cell sorter from Beckman MoFlo Astrios (Beckman Coulter, Tokyo, Japan) according to the manufacturer’s protocol, single cells were gated using the pulse height of the side scatter and forward scatter. The cells negative for FVD450 were selected as viable cells. Next, both PDPN-positive and CD31-positive cells were gated after CD45-positive cells were gated out from viable cells, namely, CD45−CD31+PDPN+ cells, which were sorted into a 2 mL tube [7]. The CD45-CD31+PDPN+ cells gated were confirmed by assessing how many cells were labeled with the anti-LYVE1 antibody (Fig 2a). The sorted cells were centrifuged, and the pellets were resuspended in 100 μL lysis buffer (Arcturus PicoPure RNA Isolation Kit, Thermo Fisher Scientific Inc.) for total RNA extraction and 200 μL of RIPA lysis buffer (EzRIPA Lysis kit, ATTO Corporation, Tokyo, Japan) for protein extraction; both the suspensions were stored at −80°C until the next step. The gated LECs were compared between the injury and sham groups 3 days after injury (*n* = 5 for each group) [24].

Total RNA (*n* = 5 for each group) was extracted from the sorted cells in lysis buffer, using an Arcturus PicoPure RNA Isolation Kit (Thermo Fisher Scientific Inc.) according to the manufacturer’s protocol. The purity of total RNA was determined using an RNA quantification kit (Thermo Fisher Scientific Inc.) and the StepOnePlus Real-Time PCR System (Thermo Fisher Scientific Inc.) according to the manufacturer’s protocol.

Proteins (*n* = 5 for each group) were extracted from the sorted cells in lysis buffer, using WSE-7420 EzRIPA Lysis kit (ATTO Corporation) according to the manufacture’s protocol. The purity of proteins was determined using the Qubit^™^ Protein and Protein Broad Range (BR) Assay Kit and Qubit 3 Fluorometer (Thermo Fisher Scientific Inc.) according to the manufacturer’s protocol.

### Microarray analysis

The GeneChip WT pico reagent kit (Thermo Fisher Scientific Inc.) was used to prepare the hybridization cocktail according to the manufacturer’s protocol. Total RNA (*n* = 3 for each group) was reverse transcribed into complementary DNA (cDNA), and complementary RNA (cRNA) was amplified *in vitro*; subsequently, single-stranded (ss)-cDNA was further transcribed from cRNA. The ss-cDNA was fragmented and labeled, and a hybridization cocktail was prepared. The cocktails were sent to Thermo Fisher Scientific Life Science Solutions for hybridization to a Clariom D Array, Mouse (Thermo Fisher Scientific Inc.) on the GeneChip instrument and were stained, washed, and scanned.

The microarray data were analyzed with Transcriptome Analysis Console (TAC) ver. 4.0 (Thermo Fisher Scientific Inc.). Gene Ontology (GO) enrichment analysis and Reactome and Kyoto Encyclopedia of Genes and Genomes (KEGG) pathway analyses were performed using g:Profiler version e105_eg52_p16_e84549f [25] or the Database for Annotation, Visualization, and Integrated Discovery (DAVID) version 6.8 [26, 27]. GO comprises three parts: biological processes (BP), molecular functions (MF), and cellular components (CC). Statistical significance was set at *p* ≤ 0.05.

A network of protein–protein interactions (PPI) was established and module analysis were performed using Cytoscape version 3.9.0 (NIGMS: National Institute of General Medical Sciences, MD, USA), attached tool Analyze Network, a plug-in of the Search Tool for the Retrieval of Interacting Genes (STRING) version 11.5 protein query, and Molecular Complex Detection (MCODE). MCODE is an automatic approach to cluster networks based on topology to determine densely connected regions [26].

### Real-time polymerase chain reaction (RT-PCR)

The cDNA (*n* = 5 for each group) was reverse transcribed with a High-Capacity cDNA Reverse Transcription Kit (Thermo Fisher Scientific Inc.) according to the manufacturer’s protocol for RT-PCR. RT-PCR was performed using the PowerUp SYBR Green PCR Master Mix (Thermo Fisher Scientific Inc.) and StepOnePlus RT-PCR System (Thermo Fisher Scientific Inc.). The primer sequences were determined using the Primer-Basic Local Alignment Search Tool (National Center for Biotechnology Information, Bethesda, MD, USA) (Table 1). The cycling parameters were as follows: 50 cycles of PCR (denaturing for 15 s at 95°C, annealing for 15 s at 60°C, and extension for 1 min at 72°C) after thermal activation for 2 min at 50°C and 95°C. Multiple housekeeping genes were used as internal standards. The ratio of mRNA expression of each target to that of peptidylprolyl isomerase A (*Ppia*), S100 protein, beta polypeptide, neural (*S100b*), and hypoxanthine guanine phosphoribosyl transferase (*Hprt*) was compared among the groups [28, 29].

**Table 1:** Sequences of the primers used for RT-PCR.

Transcript name	Primer	Sequence (5′–3′)	Accession number
*Lyve1*	Forward	TTGCCACAACTCATCCGACA	NM_053247.4
	Reverse	CTGTTGCGGGTGTTTGAGTG	
*Flt4* or *Vegfr3*	Forward	TCATTGGGGGCCTCTCCATA	NM_008029.3
	Reverse	GGCTCTCATTCGAGTGCCAT	
*Nrp2*	Forward	AAGTGGACATCCCAGAAACCC	NM_001077403.1
	Reverse	AGAGTTGCTCCAATCTCCTTCA	
*Ppia*	Forward	TTTCCGACTGTGGACAGCTCTA	NM_008907.1
	Reverse	AATGCCCGCAAGTCAAAAGA	
*Hprt*	Forward	AAGCCTAAGATGAGCGCAAGT	NM_013556.2
	Reverse	ACAGGACTCCTCGTATTTGCAG	
*S100b*	Forward	TAATGTGAGTGGCTGCGGAA	NM_009115.3
	Reverse	ATGGCTCCCAGCAGCTAAAG	

*Lyve1*: lymphatic vessel endothelial hyaluronan receptor 1. *Flt4*: FMS-like tyrosine kinase 4. *Nrp2*: neuropilin 2. *Ppia*: peptidylprolyl isomerase A. *Hprt*: hypoxanthine guanine phosphoribosyl transferase. *S100b*: S100 protein, beta polypeptide, neural.

### Enzyme-linked immunosorbent assay (ELISA)

Analysis of NRP2 protein expression (*n* = 5 for each group) was performed using the Mouse Neuropilin-2 PicoKine ELISA Kit (Boster Biological Technology, CA, USA), and FLT4 protein expression (*n* = 5 for each group) was analyzed using the Mouse VEGFR3/FLT4 PicoKine ELISA Kit (Boster Biological Technology) according to the manufacturer’s protocol. The absorbance was read using SpectraMax i3 (Molecular Device, Limited Liability Company, CA, USA) according to the manufacturer’s protocol.

### Statistical analysis

All values are reported as mean ± standard deviation. Two-group comparisons were made using the Wilcoxon-Mann-Whitney test of non-parametric statistical test. Statistical analysis was performed using JMP Pro 16, and differences were considered significant when *p* ≤ 0.05.

## Results

We removed the meninges from the CCI model TBI mice, isolated the cells, performed mRNA and protein expression analyses of CD45-CD31+PDPN+ meningeal LECs, and obtained the following results.

### Changes in the number of LYVE1-positive cells in viable meningeal LECs after TBI

**Fig 3:**
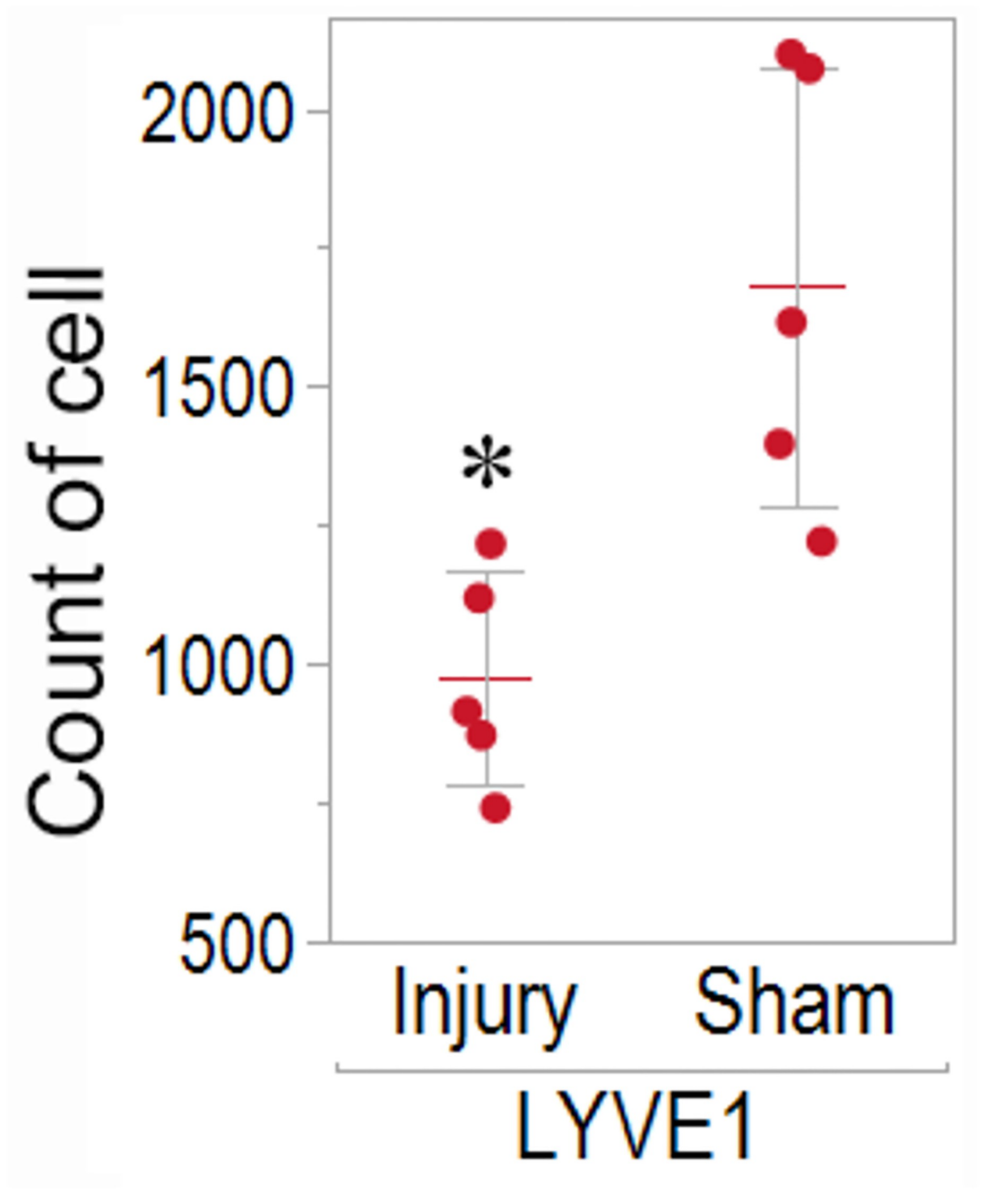
Count of viable LYVE1 positive meningeal lymphatic endothelial cells (LECs) via flow cytometry. The count of viable, CD45-CD31+PDPN+, LYVE1-positive meningeal LECs was compared between the injury and sham groups 3 days after injury via flow cytometry. (*p = 0.0122 between the injury and sham groups, *n* = 5 for each group).

We determined whether there was a change in the number of mouse meningeal LECs after TBI. The meningeal LECs (*n* = 5 for each group) were gated via flow cytometry, and the number of LECs in the injury group was compared with that in the sham group 3 days after injury (Table 2). The number of viable meningeal LECs labeled with an anti-LYVE1 antibody was significantly lower in the injury group than in the sham group (*p* = 0.0122) (Fig 3). In addition, 87.16% of all LECs were viable cells, of which, an average of 99.46% viable meningeal LECs were LYVE1 positive (Table 2).

**Table 2:** Average number of each cell gated by flow cytometry.

	Total cells	Viable cells	LECs	Viable LECs	Viable LECs/LECs	LYVE1+ cells	LYVE1+ cells/ viable LECs
Injury	237748±7822	109661±9346	1121±216*	979±193*	87.3±1.99%	973±193*	99.33±0.32%
Sham	240580±4906	110656±11126	1932±404	1688±396	87.02±2.53%	1681±395	99.59±0.21%

Viable cells: negative cells labeled with Fixable Viability Dye. LECs (lymphatic endothelial cells): CD45-CD31+PDPN+ cells (both PDPN-positive and CD31-positive cells were gated after CD45-positive cells, that is LECs, were gated out). Viable LECs: FVD-CD45-CD31+PDPN+ cells (FVD-negative, CD45-negative, CD31-positive, and PDPN-positive cells). LYVE1+ cells: positive viable LECs labeled with anti-LYVE1-antibody. (*n* = 5 for each group, *: *p* ≤ 0.05 between the injury and sham group, value: mean ± standard deviation).

### TBI-induced changes in comprehensive gene expression in meningeal LECs

To investigate the molecular biological changes in gene expression in meningeal LECs following TBI (*n* = 3 for each group), we analyzed comprehensive gene expression using microarrays.

**Fig 4:**
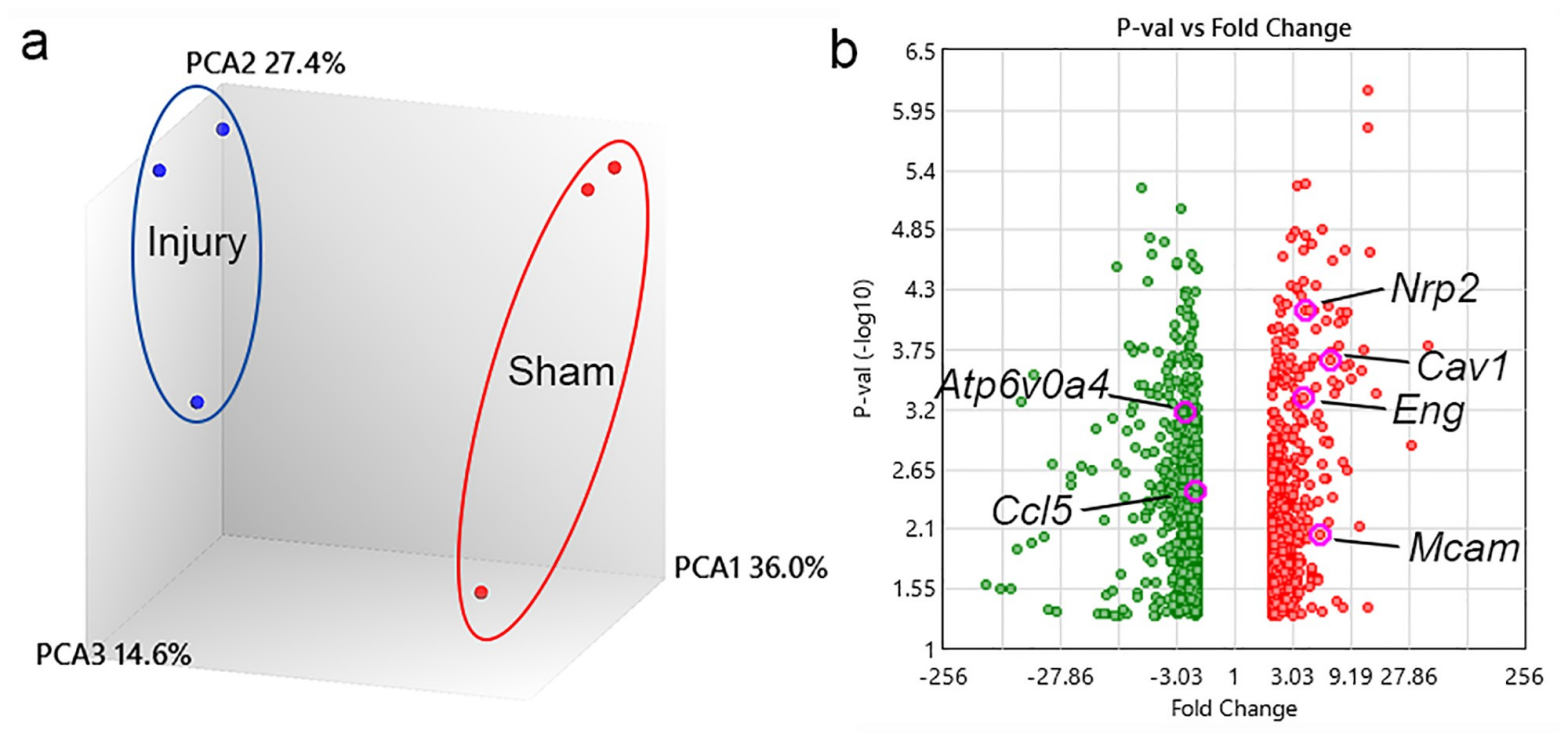
Principal component analysis (PCA) and volcano plots of the microarray data. (a) PCA shows clustering of samples. (b) The volcano plots illustrate the number of significantly differentially expressed transcripts (fold change ≥ 2 and *p* ≤ 0.05). Significantly downregulated transcripts are represented by green dot plots and significantly upregulated transcripts by red dot plots. Several transcripts with altered expression are indicated. (*n* = 3 for each group).

After 3 days of injury, principal component analysis showed that the samples within either of the groups, the injury or sham group, clustered closely together; however, the groups themselves clustered separately (Fig 4a). Compared with the sham group, the injury group showed considerable changes in the expression of differentially regulated transcripts (Fig 4b volcano plots). In the injury group, a total of 1,242 transcripts, including 617 upregulated and 625 downregulated, were found to have greater than or equal to twofold expression changes (*p* ≤ 0.05) compared to those in the sham group (Fig 4b).

#### Pathway analysis and gene ontology enrichment

The pathway and GO analyses were performed using g:Profiler with the 657 genes in the 1,242 transcripts with greater than twofold expression change, and *p* ≤ 0.05 was set as the threshold.

**Fig 5:**
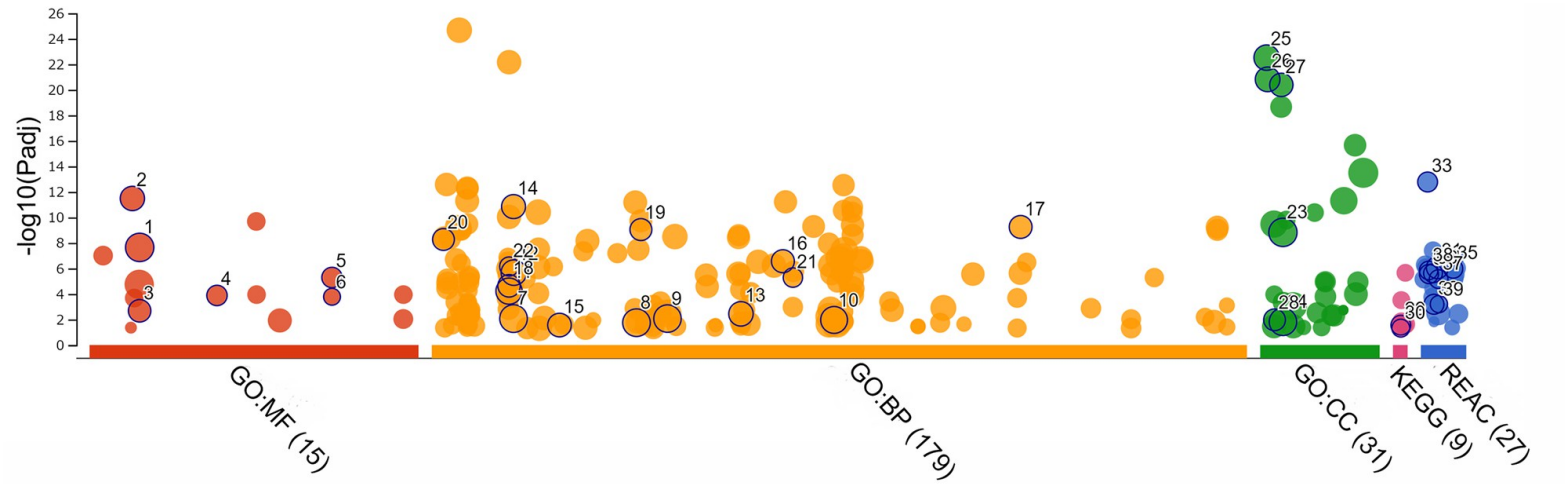
Pathways and gene ontology (GO) analyses using g:Profiler. Pathway and GO analyses of significantly differentially expressed genes (fold change ≥ 2 and *p* ≤ 0.05) are shown. The numbers identified in Fig 4 are further described in Table 3. (MF: molecular functions. BP: biological processes. CC: cellular components, REAC: Reactome pathway. KEGG: Kyoto Encyclopedia of Genes and Genomes pathway. *n* = 3 for each group).

The significant pathways and GO terms that were identified are shown in Fig 5 and Table 3, and the details are shown in S1 Table. In the pathway analysis, the selected genes were enriched in the following Reactome (R) pathways: hemostasis, Fc epsilon receptor (FCERI) signaling, cell surface interactions at the vascular wall, regulation of complement cascade, FCERI mediated mitogen-activated protein kinase (MAPK) activation, integrin cell surface interactions and B cell receptor signaling pathway, complement cascade, initial triggering of complement and classical antibody-mediated complement activation; and in the KEGG pathways: focal adhesion and extracellular matrix (ECM)-receptor interaction pathway (Table 3).

**Table 3:** Important terms obtained in gene ontology (GO) and pathway analyses via g:Profiler for 657 genes in 1242 transcriptswith ≥ 2-folds changes and P-values ≤ 0.05.

ID	Source	Term_name	Term_id	Adjusted_p_value
1	GO:MF	Protein binding	GO:0005515	2.24E-08
2	GO:MF	Signaling receptor binding	GO:0005102	3.26E-12
3	GO:MF	Calcium ion binding	GO:0005509	0.002079
4	GO:MF	Carbohydrate binding	GO:0030246	0.000130
5	GO:MF	Cell adhesion molecule binding	GO:0050839	5.08E-06
6	GO:MF	Extracellular matrix binding	GO:0050840	0.000165
7	GO:BP	Cell communication	GO:0007154	0.008487
8	GO:BP	Signaling	GO:0023052	0.017788
9	GO:BP	Developmental process	GO:0032502	0.008460
10	GO:BP	System development	GO:0048731	0.010808
11	GO:BP	Response to stress	GO:0006950	5.95E-05
12	GO:BP	Cell surface receptor signaling pathway	GO:0007166	2.12E-06
13	GO:BP	Regulation of cell population proliferation	GO:0042127	0.003600
14	GO:BP	Cell adhesion	GO:0007155	1.42E-11
15	GO:BP	Positive regulation of gene expression	GO:0010628	0.027309
16	GO:BP	Innate immune response	GO:0045087	2.70E-07
17	GO:BP	Cell-cell adhesion	GO:0098609	5.59E-10
18	GO:BP	Inflammatory response	GO:0006954	2.28E-05
19	GO:BP	Positive regulation of cell migration	GO:0030335	9.01E-10
20	GO:BP	Angiogenesis	GO:0001525	5.34E-09
21	GO:BP	Positive regulation of angiogenesis	GO:0045766	5.19E-06
22	GO:BP	Complement activation, classical pathway	GO:0006958	7.06E-07
23	GO:CC	Membrane	GO:0016020	1.49E-09
24	GO:CC	Integral component of membrane	GO:0016021	0.015166
25	GO:CC	Extracellular region	GO:0005576	3.01E-23
26	GO:CC	Extracellular space	GO:0005615	1.52E-21
27	GO:CC	Cell surface	GO:0009986	4.09E-21
28	GO:CC	Cell-cell junction	GO:0005911	0.010552
29	KEGG	Focal adhesion	KEGG:04510	0.027728
30	KEGG	ECM-receptor interaction	KEGG:04512	0.047513
31	REAC	Hemostasis	REAC:R-MMU-109582	8.55E-07
32	REAC	Fc epsilon receptor (FCERI) signaling	REAC:R-MMU-2454202	0.000640
33	REAC	Cell surface interactions at the vascular wall	REAC:R-MMU-202733	1.67E-13
34	REAC	Complement cascade	REAC:R-MMU-166658	2.72E-06
35	REAC	Regulation of complement cascade	REAC:R-MMU-977606	1.04E-06
36	REAC	FCERI mediated MAPK activation	REAC:R-MMU-2871796	2.38E-06
37	REAC	Initial triggering of complement	REAC:R-MMU-166663	6.82E-06
38	REAC	Classical antibody-mediated complement activation	REAC:R-MMU-173623	1.42E-06
39	REAC	Integrin cell surface interactions	REAC:R-MMU-216083	0.000622

MF: molecular functions. BP: biological processes. CC: cellular components. REAC: Reactome pathway. KEGG: Kyoto Encyclopedia of Genes and Genomes pathway. *n* = 3 for each group.

In the BP group, the selected genes were mostly associated with cell communication, signaling, developmental process, system development, response to stress, cell surface receptor signaling pathway, cell adhesion, angiogenesis, positive regulation of cell migration, inflammatory response, positive regulation of gene expression, cell–cell adhesion, regulation of cell population proliferation, positive regulation of angiogenesis, innate immune response, and complement activation, classical pathway (Table 3).

In the CC group, most of the selected genes exhibited a relationship with the membrane, integral component of membrane, extracellular space, extracellular region and cell surface, and cell-cell junction (Table 3).

In the MF group, the selected upregulated genes were associated with protein binding, signaling receptor binding, calcium ion binding, extracellular matrix binding, cell adhesion molecule binding, and carbohydrate binding (Table 3).

#### PPI network and MCODE analysis

**Fig 6:**
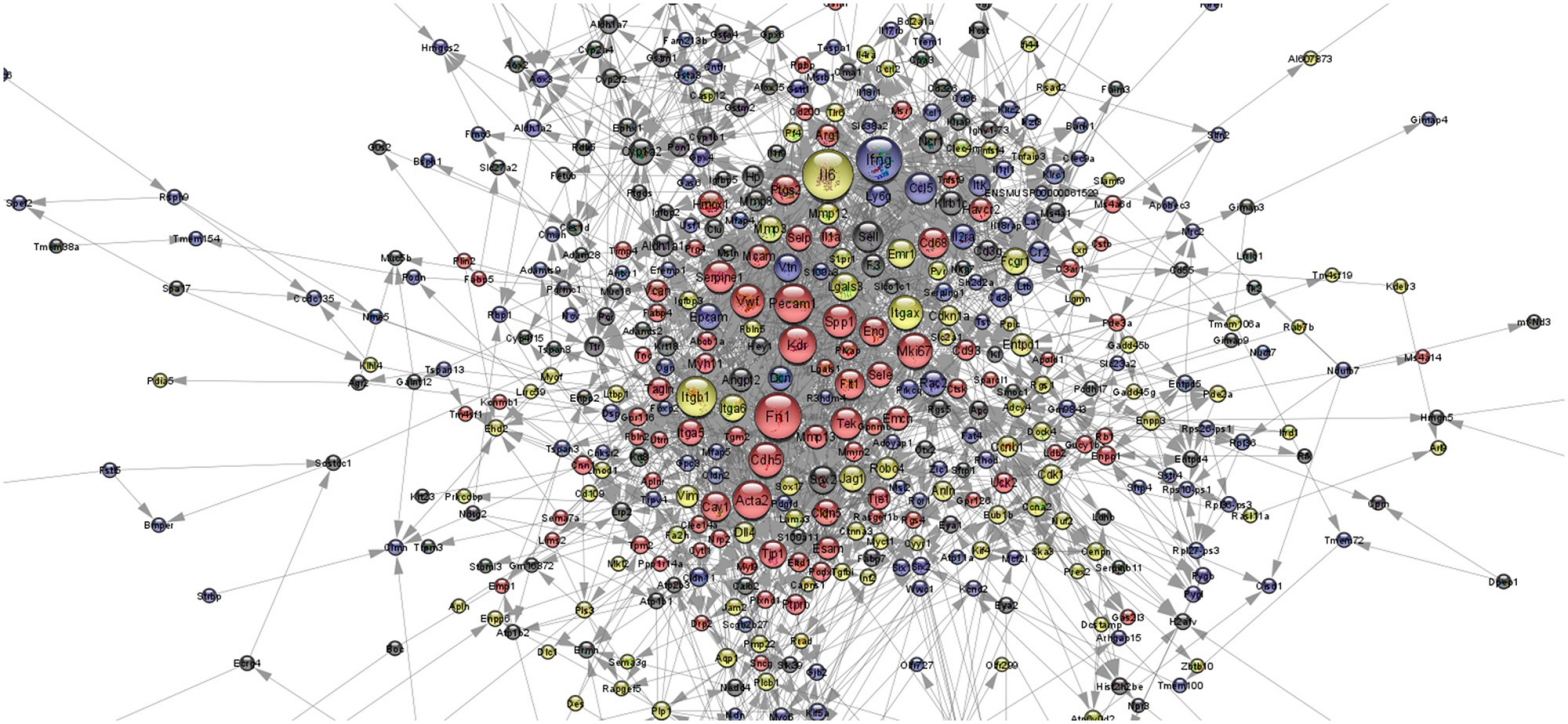
Interaction degree of the protein–protein interaction (PPI) network assessed via Cytoscape using the Search Tool for the Retrieval of Interacting Genes (STRING) plug-in. The interaction degree of the PPI network is shown based on significantly differentially expressed genes (fold change ≥ 2 and *p* ≤ 0.05). Near red color indicates upregulation, and near black color indicates downregulation; the larger the circle, the larger the value of interaction degree. (*n* = 3 for each group).

To determine the functional relationship between protein-protein interactions, 657 genes in 1,242 transcripts with greater than or equal to twofold changes and *p* ≤ 0.05 were imported into Cytoscape with the STRING plug-in. The PPI network constructed among proteins showed the associations of 657 nodes with 2,622 edges and identified candidate genes with an interaction degree ≥ 13 in the PPI network of up- and downregulated genes, respectively (Fig 6, S2 and S3 Tables). The following were recognized as the most highly ranked hub genes: *Il6* (degree 101), *Fn1* (degree 90), *Itgb1* (degree 73), *Pecam1* (degree 70), *Acta2* (degree 68), *Kdr* (degree 66), *Cdh5* (degree 58), *Itgax* (degree 57), *Vwf* (degree 55), *Spp1* (degree 53), *Serpine1* (degree 50), *Tek* (degree 49), and *Cd68* (degree 45) among the upregulated genes; *Ifng* (degree 87), *Ccl5* (degree 48), *Cyp1a2* (degree 32), *Rac2* (degree 31), *Klrb1c* (degree 27), *Ncr1* (degree 25), *Cr2* (degree 20), *Cd3g* (degree 18), *Gnal* (degree 17), *Gsta3* (degree 16), and *Ms4a1* (degree 16) among the downregulated genes (Fig 6).

**Fig 7:**
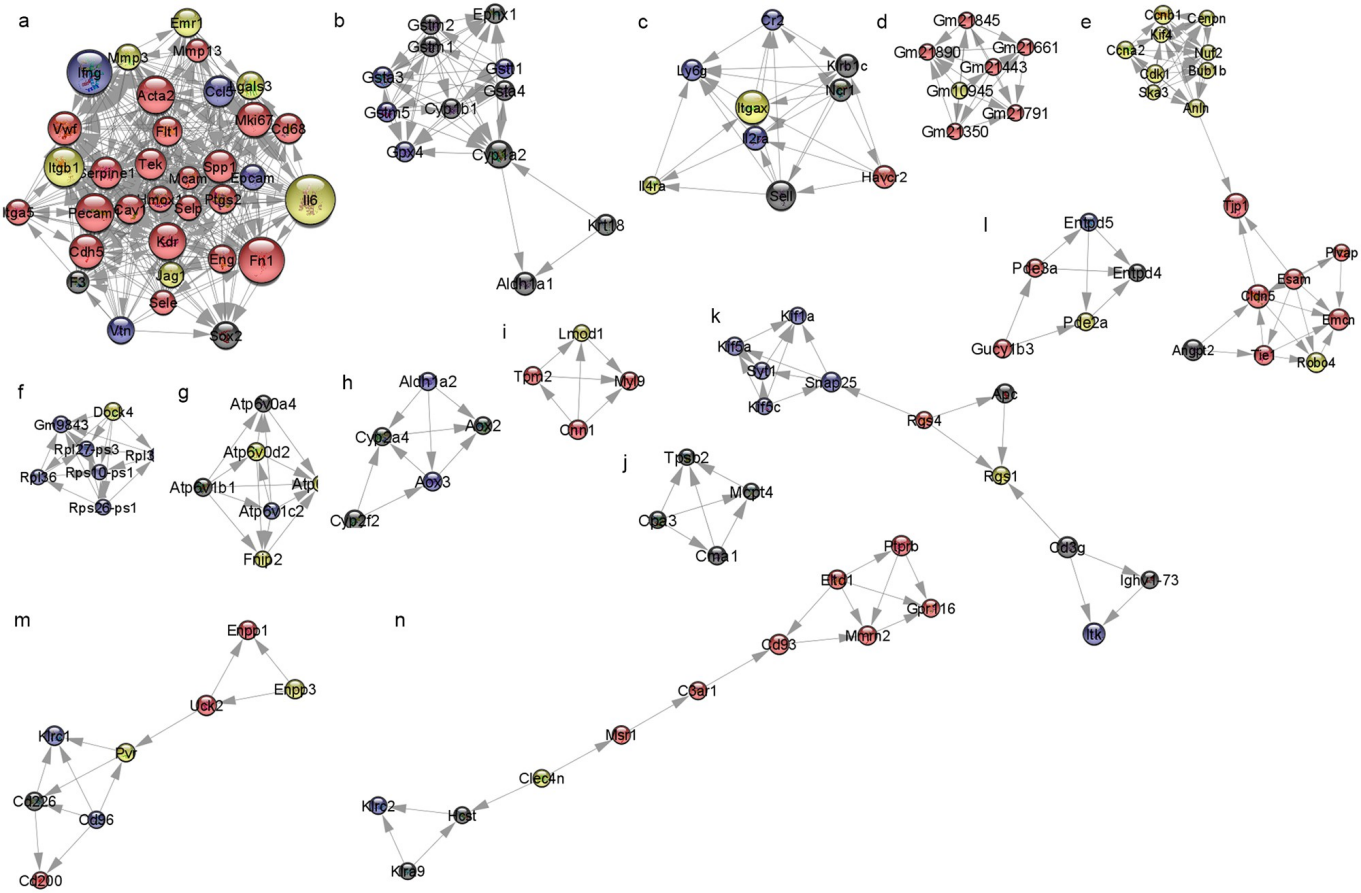
Module of molecular complex detection (MCODE) via the protein–protein interaction (PPI) network using Cytoscape and the Search Tool for the Retrieval of Interacting Genes (STRING) plug-in and MCODE tool. The module of MCODE from significantly differentially expressed genes (fold change ≥ 2 and *p* ≤ 0.05) is shown. (a–n) Modules from 1 to 14. Near red color indicates upregulation, and near black color indicates downregulation; the larger the circle, the larger the value of interaction degree. (*n* = 3 for each group).

For determining densely connected regions in the PPI network, the MCODE plug-in for Cytoscape was used for the 657 genes in 1,242 transcripts with greater than or equal to twofold changes and *p* ≤ 0.05. Modules with over four nodes were obtained (Fig 7). The GO and KEGG pathways, to which each module primarily belongs, were subjected to DAVID analysis (S4 Table). In module 1, there were 33 nodes and 378 edges (Fig 7a), which were associated with the lymphangiogenesis pathway, including the KEGG pathways mmu04151: PI3K–Akt signaling pathway, mmu04510: Focal adhesion, and mmu05323: Rheumatoid arthritis. In module 2, there were 12 nodes and 47 edges (Fig 7b), which were related to the KEGG pathways mmu00980: Metabolism of xenobiotics by cytochrome P450, mmu05204: Chemical carcinogenesis, and mmu00480: Glutathione metabolism. In module 3, there were 9 nodes and 30 edges (Fig 7c), which were related to the KEGG pathways mmu04640: Hematopoietic cell lineage and mmu04630: Jak-STAT signaling pathway. In module 4, there were 7 nodes and 21 edges (Fig 7d), which were related to the predicted gene in the Y chromosome. In module 5, there were 17 nodes and 54 edges (Fig 7e), which were related to the KEGG pathways mmu04110: cell cycle, mmu04914: progesterone-mediated oocyte maturation, and mmu04115: p53 signaling pathway. In module 6, there were 7 nodes and 20 edges (Fig 7f), which were related to translation of the structural constituent of the ribosome. In module 7, there were 6 nodes and 14 edges (Fig 7g), which were related to the KEGG pathways mmu04966: Collecting duct acid secretion, mmu04721: Synaptic vesicle cycle, and mmu05323: Rheumatoid arthritis. In module 8, there were five nodes and eight edges (Fig 7h), which were related to the KEGG pathways mmu00830: Retinol metabolism, mmu00750: Vitamin B6 metabolism, and mmu01100: Metabolic pathways. In module 9, there were four nodes and six edges (Fig 7i), which were related to the cytoskeleton. In module 10, there were four nodes and six edges (Fig 7j), which were related to the KEGG pathways mmu04614: Renin–angiotensin system. In module 11, there were 11 nodes and 18 edges (Fig 7k), which were related to the KEGG pathways mmu04721: Synaptic vesicle cycle and mmu04660: T cell receptor signaling pathway. In module 12, there were five nodes and seven edges (Fig 7l), which were related to the KEGG pathways mmu00230: Purine metabolism, mmu04022: cGMP-PKG signaling pathway, mmu04924: Renin secretion. In module 13, there were 8 nodes and 12 edges (Fig 7m), which were related to the KEGG pathways mmu00770: Pantothenate and CoA biosynthesis, mmu00500: Starch and sucrose metabolism, and mmu00760: Nicotinate and nicotinamide metabolism. In module 14, there were 11 nodes and 15 edges (Fig 7n), which were related to the integral components of the membrane.

#### Lymphangiogenesis pathway

**Fig 8:**
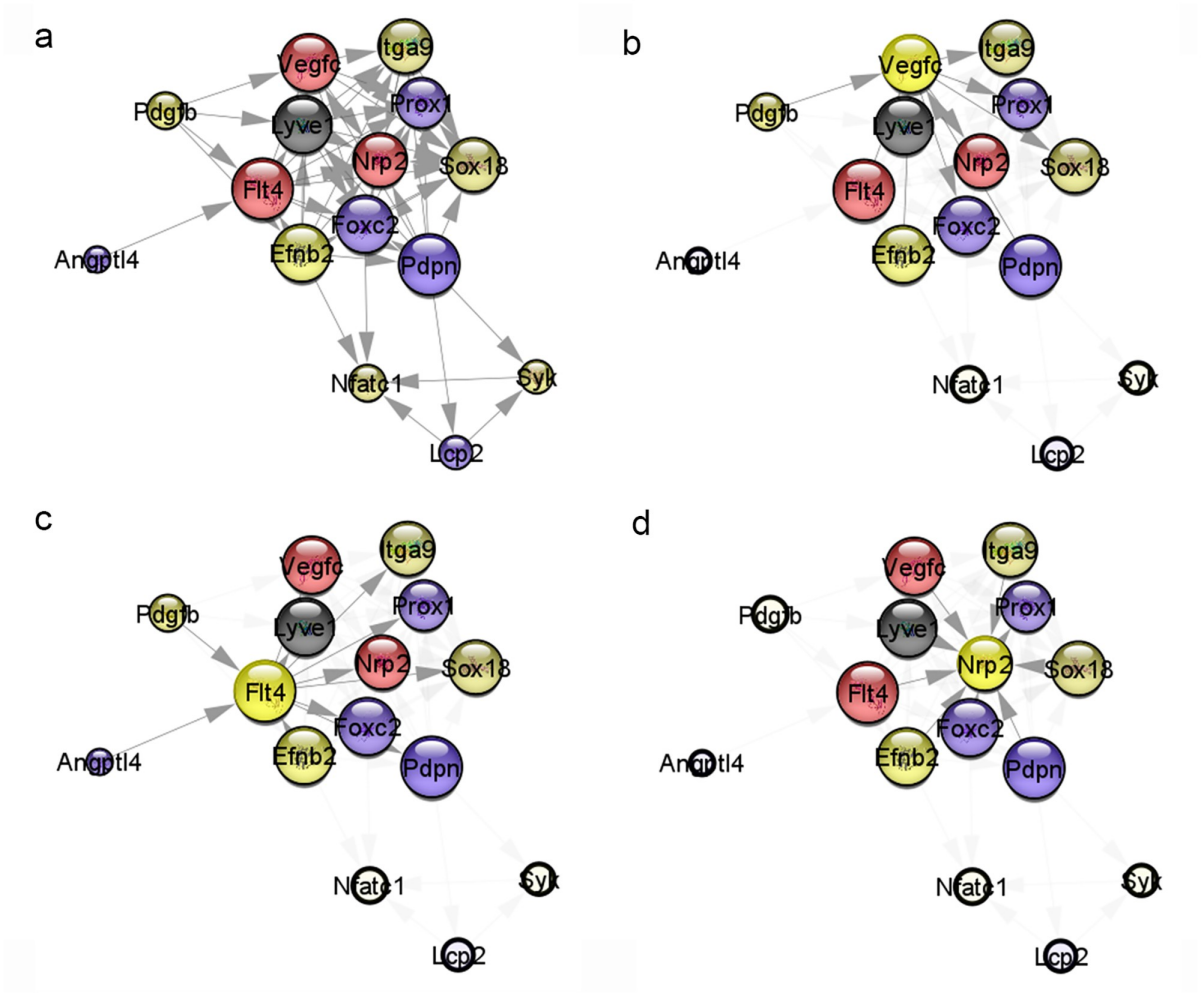
Lymphangiogenesis pathway gene of the protein–protein interaction (PPI) network obtained using Cytoscape with the Search Tool for the Retrieval of Interacting Genes (STRING) plug-in. (a) The PPI network for all lymphangiogenesis pathway genes is shown. The PPI network of genes regulating or regulated by (b) *Vegfc*, (c) *Flt4*, and (d) *Nrp2*. Near red color indicates upregulation, and near black color indicates downregulation; the larger the circle, the larger the value of interaction degree. (*n* = 3 for each group).

**Fig 9:**
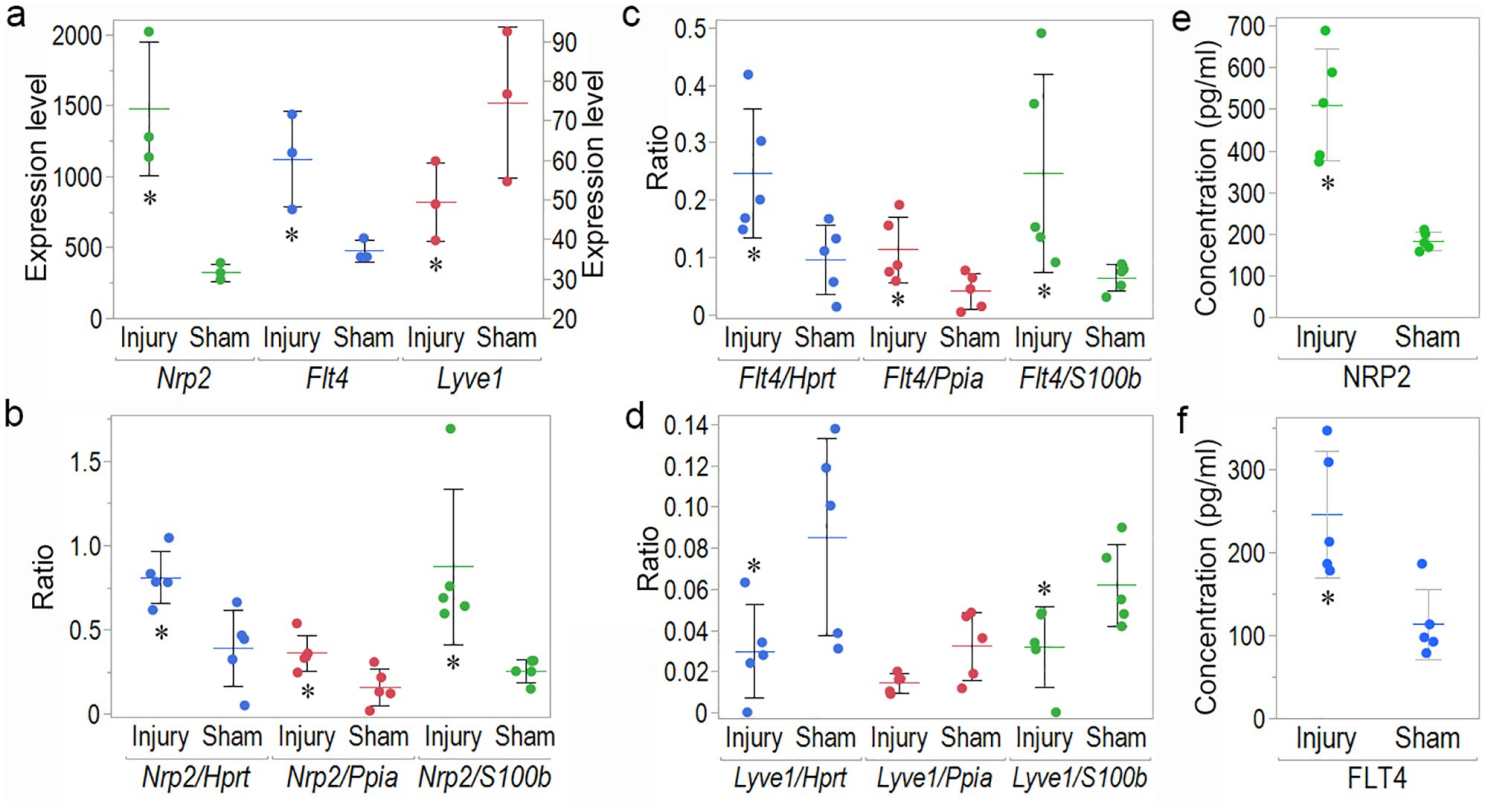
Microarray, RT-PCR and ELISA data for meningeal LECs from mice with injury and sham mice 3 days after injury. (a) The mRNA expression levels of *Nrp2* (left Y-axis), *Flt4* (right Y-axis), and *Lyve1* (right Y-axis) were determined using microarrays. The mRNA expression levels of (b) *Nrp2*, (c) *Flt4*, and (d) *Lyve1* were normalized to those of that of *Hprt*, *Ppia*, and *S100b* using RT-PCR. The protein expression levels of (e) NRP2 and (f) FLT4 in meningeal LECs, as determined using ELISA, were compared between the injury group and the sham group 3 days after injury. (*p < 0.05 between injury and sham group, *n* = 3 for each group, as assessed using microarrays, *n* = 5 for each group, as assessed using RT-PCR and ELISA).

The gene list of the lymphangiogenesis pathway based on previous studies [13, 15] was imported into Cytoscape with the STRING plug-in. The PPI network constructed among proteins showed the associations of 20 nodes with 50 edges (Fig 8a). The network analysis results are presented in Table 4. *Vegfc*, *Flt4*, and *Nrp2*, which are expressed in the early stage of lymphangiogenesis, were upregulated more than 1.5-fold in the injury group than in the sham group (Fig 9a). Apart from the expression of these three genes, the difference in the expression levels of other regulating genes and regulated genes was unchanged or decreased (Fig 8b–8d). More specifically, *Lyve1* expression in meningeal lymphatic vessels was significantly reduced (Fig 9a).

**Table 4:** Interaction degree of the protein-protein interaction network for the lymphangiogenesis pathway gene set. (*n* = 3 for each group).

Display name	Degree	P-value	Betweenness Centrality	Closeness Centrality	Fold Change
*Flt4*	11	0.0063	0.166025641	0.823529412	1.69
*Pdpn*	11	0.7777	0.216758242	0.823529412	-1.4
*Efnb2*	10	0.1965	0.074725275	0.777777778	1.46
*Foxc2*	10	0.4918	0.053571429	0.777777778	-1.14
*Lyve1*	10	0.0485	0.023168498	0.777777778	-1.53
*Vegfc*	10	0.0246	0.023168498	0.777777778	1.88
*Itga9*	9	0.4236	0.001373626	0.736842105	1
*Nrp2*	9	7.70E-05	0.001373626	0.736842105	4.14
*Sox18*	9	0.2987	0.001373626	0.736842105	1.05
*Prox1*	8	0.4779	0	0.7	-1.03
*Nfatc1*	4	0.4868	0.026373626	0.56	1.05
*Pdgfb*	4	0.0774	0	0.538461538	1.23
*Lcp2*	3	0.5966	0.002747253	0.518518519	-1.14
*Syk*	3	0.9838	0.002747253	0.518518519	1.14
*Angptl4*	1	0.6556	0	0.466666667	-1.14
*C1galt1*	1	0.3758	0	1	-1.06
*C1galt1C1*	1	0.2074	0	1	-1.15
*Spred1*	1	0.0189	0	1	1.23
*Spred2*	1	0.6823	0	1	-1.18
*Ang2*	0	0.29	0	0	-1.15
*Fndc5*	0	0.2297	0	0	-1.11

### Changes in *Lyve1*, *Flt4*, and *Nrp2* mRNA expression in meningeal LECs after TBI, determined using RT-PCR

RT-PCR for secondary screening was used to confirm the accuracy of the microarray data for primary screening. The expression of *Nrp2* and *Flt4*, which are expressed in the early stage of lymphangiogenesis, and *Lyve1*, which is specifically expressed in meningeal LECs, was examined.

*Nrp2* and *Flt4* mRNA expression increased significantly in meningeal LECs in the injury group compared to that in the sham group 3 days after injury (Fig 9b: 2.08-fold increase for *Nrp2*/*Hprt* ratio and *p* = 0.0163, 2.26-fold increase for *Nrp2*/*Ppia* ratio and *p* = 0.0163, and 3.39-fold increase for *Nrp2*/*S100b* ratio and *p* = 0.0090; Fig 9c: 2.56-fold increase for *Flt4*/*Hprt* ratio and *p* = 0.0163, 2.72-fold increase for *Flt4*/*Ppia* ratio and *p* = 0.0472, and 3.78-fold increase for *Flt4*/*S100b* ratio and *p* = 0.0090). *Lyve1* mRNA expression decreased significantly in meningeal LECs in the injury group compared to that in the sham group 3 days after injury (Fig 9d: 2.85-fold decrease for *Lyve1*/*Hprt* ratio and *p* = 0.0472, 2.25-fold decrease for *Lyve1*/*Ppia* ratio and *p* = 0.0758, and 1.92-fold decrease for *Lyve1*/*S100b* ratio and *p* = 0.0472).

### Changes in FLT4 and NRP2 protein expression in meningeal LECs after TBI

In accordance with the RT-PCR results, NRP2 (Fig 9e: 2.79-fold increase, *p* = 0.0122) and FLT4 (Fig 9f: 2.17-fold increase *p* = 0.0278) protein expression increased significantly in meningeal LECs in the injury group compared to that in the sham group 3 days after injury.

## Discussion

In this study, we observed that the number of meningeal LECs was significantly lower in the injury group than in the sham group 3 days after injury. Additionally, the mRNA expression of *Lyve1* (a specific marker of lymphatic vessels) in meningeal LECs was significantly lower in the injury group than in the sham group 3 days after injury. The mRNA and protein expression of FLT4 and NRP2 (markers of lymphangiogenesis) in meningeal LECs was significantly higher in the injury group than in the sham group 3 days after injury.

In a meningeal lymphatic drainage study, the uptake of fluorescently labeled LYVE1 antibodies in hotspots was reduced significantly compared to that in the sham group in whole-mount meninges of mice after 2 h of injury [6]. Consistent with this observation, in the present study, the number of meningeal LECs and mRNA expression of *Lyve1* in meningeal LECs was significantly lower in the injury group than in the sham group 3 days after injury.

Lymphangiogenesis is the process of LEC sprouting and proliferation as well as the differentiation of lymphatic vessels. The activation of lymphangiogenesis requires prospero-related homeobox-1 (PROX1), which functions as a transcription factor and is essential for development of the lymphatic system, as it prevents the dysfunction and rupture of lymphatic vessels. Tyrosine kinase (SYK) and the adaptor protein lymphocyte cytosolic protein 2 (LCP2) are required for the separation of blood vessels from lymphatic vessels, while angiopoietin-like 4 (ANGPTL4) is required for PROX1 expression. VEGFC signals mainly through FLT4 (VEGFR3). VEGFC/VEGFR3 signaling plays an important role in the development of lymphatic vascular pathways after the formation of the lymph sac [13]. NRP2, expressed in lymphatic vessels, can interact with VEGFR3 and bind to VEGFC. Additionally, ephrin-B2, angiopoietin-1 (ANG1), and ANG2 promote lymphangiogenesis and trigger LEC proliferation. Platelet-derived growth factor B polypeptide (PDGFB) and forkhead box C2 (FOXC2) are mainly required in the late stage of lymphatic vessel maturation [15].

Bolte et al. observed the number of capillary loops and sprouts in meningeal lymphatic vasculature morphology in TBI mice for 1 week to 2 months after injury. Additionally, it was reported that TBI induces morphological changes that occur maximally at 1–2 weeks after injury and that the lymphangiogenesis induced by mild TBI may not be permanent [6]. In this study, the mRNA expression of *Vegfc* and mRNA and protein expression of FLT4 and NRP2 in meningeal LECs, which are expressed in the early stages of lymphangiogenesis, were significantly higher in the injury group than in the sham group 3 days after injury. However, the mRNA expression of *Prox1*, *Syk*, *Lcp2*, *Angptl4*, *Pdgfb*, *ephrin-B2*, *Ang2*, and *Foxc2* was not altered. It is suggested that the mRNA expression changes during TBI-induced lymphangiogenesis may occur much earlier after injury. However, the protein expression was not sufficient for morphological observation because the other regulating or regulated genes were not expressed.

Ectonucleoside triphosphate diphosphohydrolase 1 (ENTPD1) is positively correlated with LYVE1, PDPN, and VEGFC in the LECs of patients with different cancers [30]. In this study, upregulation of *Entpd1*, located in the interaction degree of 23 of the PPI network, was found to promote lymphangiogenesis.

In module 1, wherein densely connected regions were determined in the PPI network using the MCODE plug-in of Cytoscape, the upregulated genes in LECs included interleukin 6 (*Il-6*), caveolin 1 (*Cav1*), endoglin (*Eng*), heme oxygenase 1 (*Hmox1*), integrin alpha 5 (*Itga5*), melanoma cell adhesion molecule (*Mcam*), and jagged 1 (*Jag1*) and the downregulated genes included chemokine (C-C motif) ligand 5 (*Ccl5*). IL-6 plays an important role in regulating diverse physiological and pathological processes by stimulating downstream signaling events such as the phosphatidylinositol 3-kinase (PI3K)–thymoma viral proto-oncogene 1 (Akt)–mechanistic target of rapamycin kinase (mTOR) signaling pathway and resistance to the audiogenic seizures (RAS)/MAPK pathways. In addition, MAPK signaling has been shown to contribute to various cellular functions in physiology and diseases. Therefore, activation of MAPK is required for IL-6 induction of VEGFC in LECs [31]. In this study, *Il-6* was found to exhibit the highest degree of interaction in the network, with 101 gene interactions. The IL-6-regulated MAPK signaling pathway and the PI3K–Akt–mTOR signaling pathway include CAV1, ITGA5, and IL-1 alpha (IL-1A). This result is consistent with previous research and suggests that IL-6 plays a major role in lymphangiogenesis in LECs. In addition, in prostate cancer, CAV1 affects lymphangiogenesis mediated by the panangiogenic factor vascular endothelial growth factor A (VEGFA) [32]. CAV1 inhibition results in the upregulation of CCL5 expression in human thyroid follicular epithelial cells [33]. In our study, the increase in *Vegfa* levels was greater in the injury group than in the sham group (1.43-fold change, *p* = 0.0484). The downregulation of CCL5 and lymphangiogenesis mediated by VEGFA upregulation, as indicated by our data, were possibly affected by CAV1 upregulation. ENG, a homodimeric transmembrane glycoprotein expressed in the LECs in pancreatic cancer, consists of immature endothelial cells induced by tumor lymphangiogenesis [34]. MCAM is required for the VEGFC-induced lymphatic sprouting during lymphangiogenesis [35]. ITGA5-floxed mice show defects in the lymphatic system. In addition, ITGA5 is highly expressed in lymphatic vessels and may mediate growth factor signaling, as ITGA5 is required for the optimal activation of FLT4 (VEGFR3) for proliferation and development [36]. In Kaposi sarcoma, JAG1- or delta-like canonical Notch ligand 4 (DLL4)-stimulated signaling results in the suppression of genes associated with the cell cycle in adjacent LECs, indicating a role for Notch signaling in inducing cellular quiescence in LECs [37]. HMOX1 has been reported to be associated with lymphangiogenesis [38]. Based on these observations, the upregulation of IL-6, CAV1, ENG, HMOX1, MCAM, and ITGA5, as indicated by our data, may be mediated by lymphangiogenesis or lymphatic vessel development due to meningeal lymphatic vessel injury.

Module 3 contains many genes involved in immune system processes. The upregulation of IL4 receptor alpha (IL4RA) in LECs, as observed in this study, possibly regulates the efflux of activated T cells [39].

Upregulated genes in module 5 include the genes for anillin actin binding protein (*Anln*), BUB1B mitotic checkpoint serine/threonine kinase (*Bub1b*), cyclin A2 (*Ccna2*), cyclin B1 (*Ccnb1*), cyclin-dependent kinase 1 (*Cdk1*), and endothelial cell-specific adhesion molecule (*Esam*). The p53 (transformation related protein 53)–p21 (cyclin-dependent kinase inhibitor 1A)–DREAM (DP [DP transcription factor], RB-like, E2F4 [E2F transcription factor 4], and MUVB) pathway has been shown to function via the cell cycle–dependent element (CDE) and the promoter site of the cell cycle gene homology region (CHR). In other words, switching protein binding to CDE and CHR elements regulates the downregulation of transcription by p53 associated with activation of the p53–p21–DREAM–CDE/CHR pathway [40]. G2/M cell cycle genes are possibly regulated by the p53–p21–DREAM–CDE/CHR pathway [41]. In our study, almost no change in gene expression was observed for the p53–p21–DREAM pathway, except for *p21*. The fold change in detail was −1.02 for *p53*, 2.39 for *p21*, −1.06 for *Dp*, 1.12 for *Rb*, and −1.27 for *E2f4*. BUB1B, CCNA2, and CCNB1 are G2/M cell cycle genes, and upregulation of their expression is a consequence of repression of the p53-p21-DREAM pathway [41]. Our study revealed upregulation of the genes for *Bub1b*, *Ccna2*, and *Ccnb1*, and it is suggested that these genes are associated with inactivation of the p53–p21–DREAM–CDE/CHR pathway and that they may activate the cell cycle. In addition, inflammation-associated IL-6 is one of the factors associated with the senescence-associated secretory phenotype (SASP). On the other hand, p21 is associated with cellular senescence [42]. Upregulation of IL-6 and p21 expression may be due to an inflammatory driven senescence phenotype, which may protect against cell damage. In addition, the upregulation of *Cdk1* and *Anln* in this study suggests that the CDK1-PLK1 (polo like kinase 1)/SGOL2 (shugoshin 2A)/ANLN pathway may mediate the division of LECs in the cell cycle [43]. ESAM, a regulatory tight junction protein, was upregulated in tumor LECs [44]. The upregulation of *Esam* in our study was possibly caused by meningeal lymphatic vessel injury.

Module 7 consisted of several vacuolar H+-ATPases (V-ATPases) such as ATPase H+ transporting lysosomal V0 subunit A4 (ATP6V0A4), ATP6V0D2, ATP6V0E, ATP6V1B1, and ATP6V1C2. V-ATPase, as a rotary proton pump, plays an important role in the pH regulation of cells and their intracellular compartments. V-ATPase is composed of a cytosolic V1 domain and a transmembrane V0 domain. These domains consist of multiple subunits [45]. In RNA profiling data sets generated by the Mouse Encyclopedia of DNA Elements (ENCODE) project, ATP6V0A4 is expressed in 2 tissues, ATP6V0D2 in 7 tissues, ATP6V0E in 30 tissues, and ATP6V1B1 and ATP6V1C2 in 5 tissues [46]. According to our data, in the LECs, *Atp6v0d2* and *Atp6v0e* were upregulated and *Atp6v0a4*, *Atp6v1b1*, and *Atp6v1c2* were downregulated. ATP6V0D2 and ATP6V0E exhibited the tendency to be expressed in many tissues.

It has been reported that in Kaposi sarcoma, numerous genes are expressed in LECs. However, G-protein signaling (RGS) 4 is not expressed in LECs, as Kaposi sarcoma-associated herpes virus upregulates PROX1, which is involved in LEC differentiation, leading to the downregulation of RGS4 [47]. Upregulation of the regulator of *Rgs1* and *Rgs4* may be related to no expression changes in *Prox1*, in module 11 of this study.

Ectonucleotide pyrophosphatase/phosphodiesterase 1 (ENPP1) is a member of the ENPP family. Ectonucleotidases, such as ENPP1, hydrolyze adenosine triphosphate (ATP) into adenosine, leading to anti-inflammatory effects and protection of cells and tissues from excessive inflammation and immune-mediated damage. ATP is a member of the purine signaling pathway, which is a class of biological actions mediated by specific receptors for extracellular nucleotides such as ATP and adenosine, and it plays an important role in a wide range of inflammatory conditions and inflammatory diseases. Upregulation of *Enpp1* and *Enpp3* in module 13 of our study was suggested to play an anti-inflammatory role in the inflammation induced by meningeal lymphatic vessel injury [48, 49].

Macrophage scavenger receptor 1, which was found to be upregulated in module 14 of our study, mediates the interaction between LEC and lymphocytes involved in adhesion. It has been suggested to contribute to lymphangiogenesis and cellular traffic as well as to the control of metabolism and fluid balance [50].

Regarding surgical methods in the sham group, a previous paper reported that craniotomy should not be performed because of the brain damage caused by the vibration and heat of the drill or deviation of operation [51]. In this study, we adopted a non-craniotomy method to avoid brain and meninx damage, considering that six times more mice would be sacrificed than usual because we pooled the meninges of six mice per sample. Therefore, we believe that the number of mice used in this study was reduced due to a reduction in brain and meninx damage during craniotomy, preventing the failure of the sham mouse experiment.

This study focused on the LECs involved in meningeal lymphatic vessel injury associated with TBI. However, we did not analyze how the lymphatic vessel injury affects brain function. It seems more natural to assume that the brain dysfunction caused by the brain injury itself is greater than that caused by the meningeal lymphatic vessel injury in the acute phase of TBI. After meningeal injury, meningeal lymphangiogenesis begins, and lymphatic vessel function may be restored in the short-term. However, after some time, when the brain tissue has largely repaired, lymphatic vessels that have been damaged may have a long-term effect on brain function. It will be a subject of future research to determine how lymphatic vessels that have been damaged at a young age affect brain function over time. Such research is also expected to contribute to the treatment of TBI as well as the prevention of sequelae of TBI by improving the function of meningeal lymphatic vessels.

## Conclusion

The number of meningeal LECs was significantly lower in the injury group than in the sham group 3 days after TBI. Additionally, the mRNA expression of *Lyve1* in meningeal LECs was significantly lower in the injury group than in the sham group. The mRNA and protein expression of FLT4 and NRP2 in meningeal LECs was significantly higher in the injury group than in the sham group. TBI is associated with the impairment of meningeal LECs and meningeal lymphangiogenesis, which implicates lymphatic vessel injury in the pathogenesis of this condition.

## Supporting information

S1 Table All terms in Gene Ontology (GO) and pathway analyses, determined by g:Profiler, for 657 genes in 1242 transcripts with ≥ 2-folds changes and P-values ≤ 0.05. (XLSX)

S2 Table Interaction degree of the protein-protein interaction network for the upregulated genes of 657 genes in 1242 transcripts with ≥ 2-fold changes and *p* values ≤ 0.05. (XLSX)

S3 Table Interaction degree of the protein–protein interaction network for the downregulated genes of 657 genes in 1242 transcripts with ≥ 2-fold changes and *p* values ≤ 0.05. (XLSX)

S4 Table Modules 1 to 14 assessed using the molecular complex detection (MCODE) plug-in Cytoscape and Visualization and Integrated Discovery (DAVID) for genes of 657 genes in 1242 transcripts with ≥ 2-fold changes and *p* values ≤ 0.05. GO: gene ontology, BP: biological process, CC: cellular component, MF: molecular function, and KEGG: Kyoto encyclopedia of genes and genomes.
(XLSX)

S1 Checklist (PDF)
